# Internet-Based Audiologist-Guided Cognitive Behavioral Therapy for Tinnitus: Randomized Controlled Trial

**DOI:** 10.2196/27584

**Published:** 2022-02-14

**Authors:** Eldré W Beukes, Gerhard Andersson, Marc Fagelson, Vinaya Manchaiah

**Affiliations:** 1 Vision and Hearing Research Centre Anglia Ruskin University Cambridge United Kingdom; 2 Virtual Hearing Lab, Collaborative Initiative Between University of Colorado School of Medicine and University of Pretoria Aurora, CO United States; 3 Department of Behavioral Sciences and Learning Linköping University Linköping Sweden; 4 Department of Audiology and Speech-Language Pathology East Tennessee State University Johnson City, TN United States; 5 Auditory Vestibular Research Enhancement Award Program Audiological Rehabilitation Laboratory Veterans Affairs Medical Center Mountain Home, TN United States; 6 Department of Speech-Language Pathology and Audiology University of Pretoria Pretoria South Africa; 7 Department of Otolaryngology–Head and Neck Surgery University of Colorado School of Medicine Aurora, CO United States; 8 UCHealth Hearing and Balance University of Colorado Hospital Aurora, CO United States; 9 Department of Speech and Hearing Manipal College of Health Professions Manipal Academy of Higher Education Manipal India

**Keywords:** tinnitus, cognitive behavioral therapy, internet intervention, web-based intervention, randomized controlled trial, telehealth, teleaudiology, eHealth

## Abstract

**Background:**

Tinnitus is a symptom that can be very distressing owing to hearing sounds not related to any external sound source. Managing tinnitus is notoriously difficult, and access to evidence-based care is limited. Cognitive behavioral therapy (CBT) is a tinnitus management strategy with the most evidence of effectiveness but is rarely offered to those distressed by tinnitus. The provision of internet-based CBT for tinnitus overcomes accessibility barriers; however, it is not currently readily available in the United States.

**Objective:**

The aim of this study is to investigate the efficacy of internet-based CBT compared with that of weekly monitoring for the management of tinnitus in reducing tinnitus distress; reducing tinnitus-related comorbidities, including tinnitus cognitions, insomnia, anxiety, and depression; and assessing the stability of the intervention effects 2 months after the intervention.

**Methods:**

A 2-arm randomized clinical trial comparing audiologist-guided internet-based CBT (n=79) to a weekly monitoring group (n=79) with a 2-month follow-up assessed the efficacy of internet-based CBT. Eligible participants included adults seeking help for tinnitus. Recruitment was conducted on the web using an open-access website. Participants were randomized via 1:1 allocation, but blinding was not possible. The study was undertaken by English or Spanish speakers on the web. The primary outcome was a change in tinnitus distress as measured using the Tinnitus Functional Index. Secondary outcome measures included anxiety, depression, insomnia, tinnitus cognition, hearing-related difficulties, and quality of life.

**Results:**

Internet-based CBT led to a greater reduction in tinnitus distress (mean 36.57, SD 22) compared with that in weekly monitoring (mean 46.31, SD 20.63; effect size: Cohen *d*=0.46, 95% CI 0.14-0.77) using an intention-to-treat analysis. For the secondary outcomes, there was a greater reduction in negative tinnitus cognition and insomnia. The results remained stable over the 2-month follow-up period. No important adverse events were observed. Further, 16% (10/158) of participants withdrew, with low overall compliance rates for questionnaire completion of 72.3% (107/148) at T1, 61% (91/148) at T2, and 42% (62/148) at T3.

**Conclusions:**

This study is the first to evaluate and indicate the efficacy of audiologist-delivered internet-based CBT in reducing tinnitus distress in a US population. It was also the first study to offer internet-based CBT in Spanish to accommodate the large Hispanic population in the United States. The results have been encouraging, and further work is indicated in view of making such an intervention applicable to a wider population. Further work is required to improve compliance and attract more Spanish speakers.

**Trial Registration:**

ClinicalTrials.gov NCT04004260; https://clinicaltrials.gov/ct2/show/NCT04004260

## Introduction

### Background

Tinnitus is a condition characterized by the perception of sound in the absence of an external stimulus. It is highly prevalent, with at least 10% of Americans experiencing some form of tinnitus, of which a proportion have chronic burdensome or debilitating tinnitus [1]. Bothersome tinnitus causes various functional impairments in sleep, concentration, cognitive performance, and thought processing [2,3]. It is also associated with an increased risk of psychological difficulties, such as anxiety, depression, and reduced quality of life [4,5]. This may result in participant restrictions and activity limitations [6,7]. Owing to the negative impact of tinnitus, those distressed by their tinnitus require interventions to help them cope with the condition.

Managing tinnitus is notoriously challenging because it is often not a curable medical cause [8]. When comorbid problems such as hearing loss accompany tinnitus, hearing aids and sound therapy may reduce the severity of tinnitus [8]. Although these interventions may reduce tinnitus perception, they do not always alter negative reactions to tinnitus. Psychological interventions that change reactions to tinnitus have effectively helped reduce tinnitus distress [9]. The intervention with the strongest research evidence according to the American Academy of Otolaryngology–Head and Neck Surgery tinnitus practice guidelines [10-12] and several systematic reviews [9,13] is cognitive behavioral therapy (CBT) for tinnitus. CBT is a psychological intervention that addresses unhelpful thought patterns and emotional reactions caused by tinnitus [14]. Despite the evidence base, accessibility to CBT for tinnitus is limited owing to a dearth of health care providers with the knowledge and expertise to provide CBT to this population [15,16].

### Previous Work

To overcome this barrier, an internet-based CBT for tinnitus [17] has been developed. This intervention was originally developed in Swedish [18] and was later translated into German [19] and English [20] and was provided with psychological guidance. To further increase accessibility, internet-based CBT for tinnitus has been adapted to be delivered by audiologists [21] with some training to handle the CBT elements without compromising outcomes [22-27]. The efficacy of internet-based CBT has been indicated in 9 clinical trials across mainland Europe and the United Kingdom (for a review, see the study by Beukes et al [28]). However, no clinical trials determined the effects of internet-based CBT in the United States. An evidence-based, standardized approach, such as internet-based CBT, is desirable, as tinnitus provision varies substantially across clinics and providers [16].

### Study Rationale

To address this need, internet-based CBT was adapted for the US population to improve cultural and linguistic suitability [29]. It was further translated into Spanish to serve the large Spanish-speaking population in the United States, and functionality and acceptability testing was undertaken [30]. Internet-based CBT for tinnitus in the United States was evaluated within a clinical trial framework to evaluate complex interventions [31]. A small (N=28) phase I trial was undertaken in the United States [32], indicating feasibility. However, a larger randomized controlled trial (RCT) is needed to determine its efficacy in the US population. Efficacy cannot be assumed owing to many differences in health care provision in the United States and Europe. In the United States, tinnitus interventions include Tinnitus Retraining Therapy [33] and Progressive Tinnitus Management [34] with limited provision of CBT for tinnitus. It is also not known if a psychological approach will be acceptable because of the large emphasis of most tinnitus management programs on sound therapy and the fitting of devices [35]. The fee structure for health care in the United States is also very different to largely free of charge health care in the United Kingdom, as it is generally paid out of pocket, and clinicians have great difficulty receiving payment for nondiagnostic appointments such as tinnitus counseling. Before the COVID-19 pandemic, health care was also generally provided face to face. Hence, the uptake for a remote intervention is uncertain but has now become a more urgent matter. These factors may be potential barriers to or facilitators of internet-based CBT in the United States.

This RCT aims to explore the effects of internet-based CBT in the United States with the following:

1. To evaluate the efficacy of audiologist-delivered internet-based CBT in reducing tinnitus distress compared with that in weekly monitoring of tinnitus.
2. To ascertain the efficacy of internet-based CBT in reducing comorbidities associated with tinnitus.
3. To assess the stability of internet-based CBT intervention effects 2 months after the intervention.

The hypothesis was that patients with tinnitus would experience greater reduction of tinnitus distress and comorbidities after receiving internet-based CBT compared with patients receiving weekly monitoring.

## Methods

### Trial Design

#### Overview

**Figure 1:**
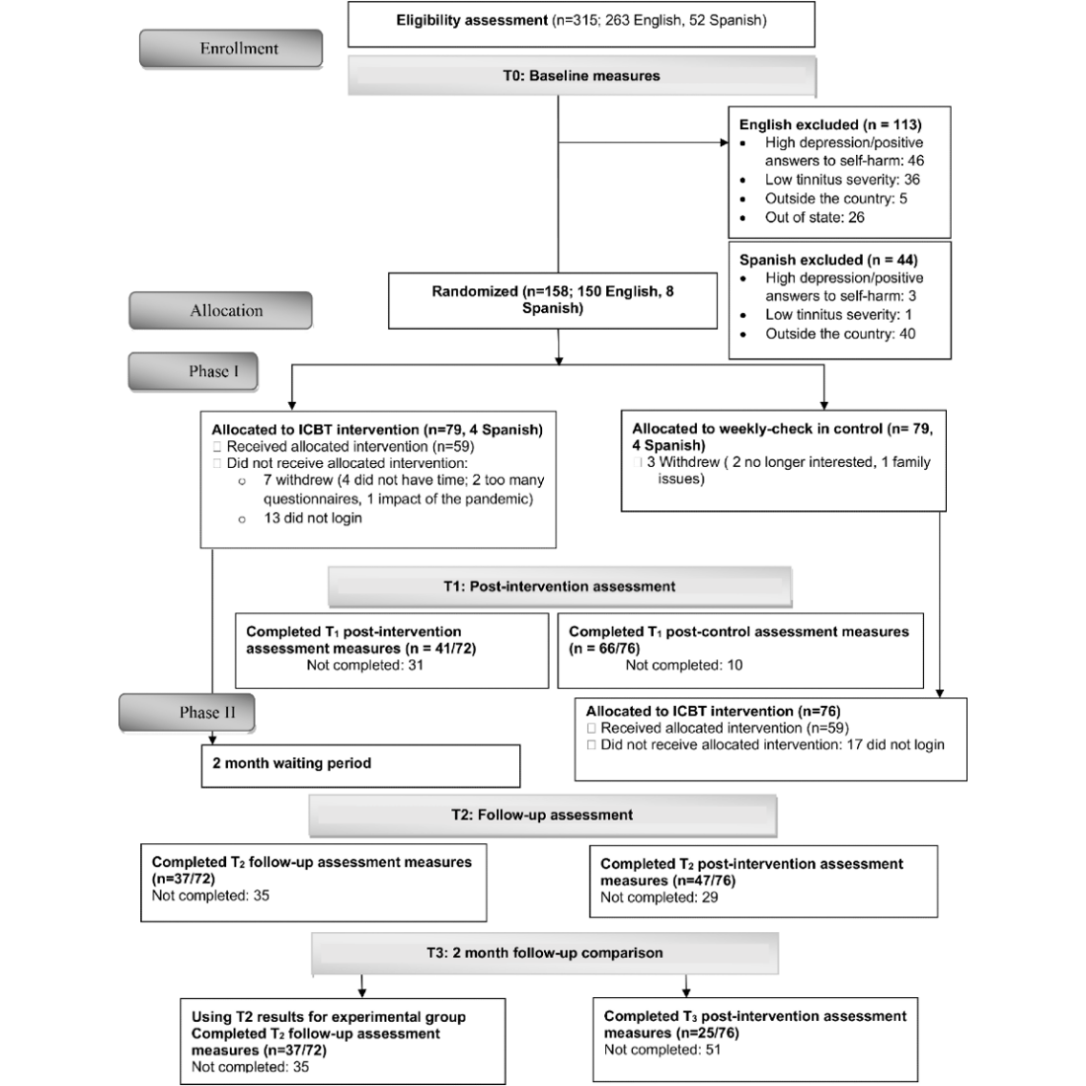
The CONSORT (Consolidated Standards of Reporting Trials) flow diagram. ICBT: internet-based cognitive behavioral therapy.

A prospective 2-arm delayed intervention efficacy trial with a 2-month follow-up was conducted. As an efficacy trial, an active comparator was not included. Participants were randomized with a 1:1 allocation ratio to the experimental group to receive the internet-based CBT intervention for 8 weeks or the control group whose participants were monitored weekly during the 8-week period. During the first phase, the experimental group completed the intervention. Following the experimental group completing the interventions, both groups completed the same outcome measure (T1, post intervention). During the second phase, the control group underwent the same internet-based CBT intervention, after which both groups were invited to complete the T2 outcomes, which were completed after the control group finished the intervention and 2-months postintervention for the experimental group (T2). This study design, therefore, provided the opportunity to evaluate the intervention effects in 2 independent groups at 3 time points as shown in Figure 1.

#### Ethics and Preregistration

This RCT and protocol were preregistered at ClinicalTrials.gov (trial number: NCT04004260) on July 2, 2019. Ethical approval was obtained from the Institutional Review Board at Lamar University, Beaumont, Texas, United States (IRB-FY17-209). The study was conducted and reported according to the CONSORT-EHEALTH (Consolidated Standards of Reporting Trials of Electronic and Mobile Health Applications and Online Telehealth) guidelines [36] (Multimedia Appendix 1). An independent data-monitoring committee monitored the trial. There were no changes in the methods or trial outcomes used after the trial commenced. The participants were closely monitored for any harm. No harm or unintended effects have been reported.

#### Participants

The study was undertaken on the web and no clinical visits were required. The study eligibility criteria are presented in Textbox 1.

**Inclusion and exclusion criteria.**

**Inclusion criteria**

- Adults, aged ≥18 years, living in Texas, United States
- The ability to read and type in English or Spanish
- Having access to a computer and the internet and able to email
- Experiencing tinnitus for a minimum period of 3 months
- A tinnitus severity score of ≥25 on the Tinnitus Functional Index indicates the need for an intervention

**Exclusion criteria**

- Indications of significant depression (≥15) on the Patient Health Questionnaire-9
- Indications of self-harm thoughts or intent, answer affirming question 10 of the Patient Health Questionnaire-9
- Reporting any medical or psychiatric conditions that could interfere with the treatment
- Reporting pulsatile, objective, or unilateral tinnitus, which has not been investigated medically or tinnitus still under medical investigation
- Undergoing any tinnitus therapy concurrent with participation in this study

Eligibility was determined by a 2-stage process as follows:

1. A web-based screening questionnaire, which included demographic information, health- and mental health–related questions, and standardized outcome measures are shown in Table 1.
2. A telephone interview during which the researcher rechecked eligibility and provided the opportunity for potential participants to ask any questions related to the study. The study procedures were explained, and motivational interviewing was conducted to encourage participants to commit and engage in the intervention.
3. Participants who did not meet the inclusion criteria were participants with a score of ≥15 on the Patient Health Questionnaire-9 or indicated self-harm on question 10 received a phone consultation from a clinical psychologist on the research team. This call ensured that they were under care elsewhere, or necessary resources or referrals were provided.

**Table 1:** Study outcome measures used before and after the intervention and at the 2-month follow-up period.

Dimension		Outcome measures	Internal consistency	Scores (range)	Levels of significance	Time frame measured	
**Primary outcome measure**							
	Tinnitus distress	Tinnitus Functional Index: 42	0.97	0-100; a reduction in scores indicates improvement	>25 indicates a mild condition (no need for an intervention), 26-50 indicates a significant condition (possible need for an intervention), and ≥50 indicates a severe condition (need for a more intense intervention)	T0^a^, T1^b^, T2^c^, and T3^d^ (control only)	
**Secondary outcome measures**							
	Generalized anxiety	Generalized Anxiety Disorder-7: 44	0.89	0-21; a reduction in scores indicates improvement	0-4 indicates minimal anxiety, 5-9 indicates mild anxiety, 10-14 indicates moderate anxiety, and 5-21 indicates severe anxiety	T0, T1, T2, and T3 (control only)	
	Depression	Patient Health Questionnaire-9: 45	0.83	0-27; a reduction in scores indicates improvement	5-9 indicates mild depression, 10-14 indicates moderate depression, 15-19 indicates moderately severe depression, and 18-20 indicates severe depression	T0, T1, T2, and T3 (control only)	
	Insomnia	Insomnia Severity Index: 46	0.74	0-28; a reduction in scores indicates improvement		T0, T1, T2, and T3 (control only)	
	Tinnitus cognitions	Tinnitus Cognitions Questionnaire: 47	0.91	0-104; a reduction in scores indicates improvement	Higher scores indicate a greater tendency to engage in negative cognitions in response to tinnitus	T0, T1, T2, and T3 (control only)	
	Health-related quality of life	EQ-5D-5L^e^: 48	0.7-0.85	0-15; a reduction in scores indicates improvement	Measures 5 dimensions: mobility, self-care, usual activities, pain, discomfort, anxiety and depression	T0, T1, T2, and T3 (control only)	
	Health-related quality of life	EQ-5D-5L visual analog scale: 48	0.7-0.85	0-100; higher scores indicate improved health	Visual analog scale for overall health	T0, T1, T2, and T3 (control only)	
	Short measure for tinnitus, hearing disability, and hyperacusis	Tinnitus and Hearing Survey: 49	0.86-0.94	Subscale for tinnitus: 0-16; subscale for hearing: 0-16; subscale for sound tolerance: 0-8	—^f^	T0, T1, T2, and T3 (control only)	
**Weekly monitoring**							
	Screening of tinnitus severity	Tinnitus Handicap Inventory–Screening [37]	0.93	0-40; a reduction in scores indicates improvement	>6 indicates tinnitus handicap	Weekly, while undertaking the 8-week intervention	
	Tinnitus perception	Tinnitus Qualities Questionnaire [21]	Not assessed	0-100; a reduction in scores indicates improvement	Designed to determine whether tinnitus qualities such as loudness, pitch, the number of tones heard, and so forth improves while undertaking an intervention. Higher scores indicate that more bothersome aspects of tinnitus are present.	Weekly, while undertaking the 8-week intervention	

^a^T0: before intervention.

^b^T1: 8 weeks after the experimental group started the intervention (before the control group started the intervention).

^c^T2: 8 weeks after the control group completed the intervention (2 months after the experimental group completed the intervention).

^d^T3: 2 months after the control group completed the intervention.

^e^EQ-5D-5L: European Quality of Life Five Dimension.

^f^Higher scores indicate more problematic tinnitus, hearing disability, and hyperacusis.

#### Recruitment Strategy

In line with the US government’s health promotion initiative to make health care linguistically and culturally accessible [38], all the study materials were available in both English and Spanish and were also written at or below the sixth-grade English reading level [29,39]. The participants were mostly recruited from the general public using a range of strategies including a television broadcast, promoting the study via tinnitus support groups in Texas and the American Tinnitus Association, and contracting the company TrialFacts to boost recruitment. Further recruitment strategies included the use of social media (eg, Facebook and Twitter), flyers, and posters, which were distributed to local communities and put up in clinic waiting rooms. Professionals such as audiologists and otolaryngologists in Texas were also notified about the study and provided with leaflets to distribute to suitable patients. Recruitment was conducted on the web from an open access website between February 17, 2020, and March 30, 2020. Those interested were directed to the study website, which had detailed information about the study and the study team and registered their interest in study participation. Informed consent was provided on the web, confirming the understanding of how data would be used, to be randomized, the length of the trial, the commitment expected, and being contacted for follow-up data collection. Following registration, they were invited to complete the web-based screening, demographic, and outcome questionnaire. They were informed of their right to withdraw without penalty at any stage of the process.

#### Sample Size, Power, and Attrition

Sample size estimation was calculated using G*Power (version 3.1.6) [40] and based on achieving a 13-point clinically meaningful change between the baseline and postintervention measurements using the primary assessment measure, the Tinnitus Functional Index (TFI). Pilot data [32] indicated 26 participants per group with a 1:1 allocation to achieve 80% power to detect a between-group mean standardized difference effect size of Cohen *d*=0.50 (a moderate effect size). As this was fewer than the 58 participants suggested using data from an internet-based CBT RCT from the United Kingdom [23], we selected 58 participants per group. In addition, the sample size was inflated to account for missing data, estimated to be 20% of the US phase I trial data [32]. Thus, 146 participants were recruited, with 73 in each arm (calculated as 58/0.8).

#### Randomization

Participants meeting the inclusion criteria were randomly assigned in a 1:1 ratio and enrolled in either the experimental or control group using a computer-generated randomization schedule by an independent research assistant in blocks of varying sizes after participants were prestratified for language (English and Spanish). Participants and investigators could not be blinded to the group allocation owing to the nature of the intervention. Participants were informed immediately after randomization when the intervention commenced by the principal investigator, but not explicitly to which group they were assigned.

#### Patient-Public Partnership

A patient-public partnership was established to include 2 individuals with tinnitus who had piloted the internet-based CBT intervention, 2 audiologists, and 2 researchers. The meetings were held via videoconferencing. The aim of the patient-public partnership was to guide the study processes and provide inputs to the research strategy to boost recruitment and other elements of the study to ensure high compliance and engagement

### Intervention

The internet-based CBT intervention content was based on a CBT self-help program originally developed in Swedish [18] and later adapted and translated into English [20]. The intervention was subsequently transformed into an 8-week interactive e-learning version suitable for the UK population [41] and then adapted linguistically and culturally to ensure suitability for the US population [29]. These adaptions prioritized accessibility of the intervention, such as lowering the readability to below the recommended sixth-grade level, as more than half of the US adult population has low literacy skills [42]. The intervention was augmented by a mindfulness module and more videos. The internet-based CBT platform (version 1) in the US application consisted of 22 modules with worksheets and quizzes (see [21,29] for more details). Participants required an internet connection to access the materials and email correspondence regarding the intervention.

The intervention platform was transferred from Sweden and housed in the United States (Lamar University) to comply with the required US data protection regulations. Before this feasibility trial, the acceptability and functionality of this intervention for the US population were first ensured [30].

Both groups received the same intervention, and only the timing of receiving the intervention varied. The control group received the experimental intervention 8 weeks after the experimental group commenced the program. The intervention was 8 weeks long. Participants were asked to read the modules weekly and ideally spent 10 minutes each day practicing the suggested strategies.

### Audiology Guidance

Guidance was provided to support the participants while undertaking the intervention. This included monitoring progress, monitoring weekly scores, providing feedback on worksheets completed, outlining the content of new modules, and answering questions. Participants who did not engage were contacted to support their participation and to discuss possible barriers. An encrypted 2-way messaging system was used to communicate with a minimum guidance time of 10 minutes per participant. Although psychologists have traditionally provided CBT interventions, audiologists generally deliver tinnitus management [16]. Thus, audiologists provided guidance to participants in a manner consistent with previous English trials [22-24] using this intervention to ensure standardization of the intervention approach. Support to the Spanish participants was provided by a Doctor of Audiology student with clinical experience whose first language was Spanish, using a handbook that was developed by the lead English-speaking audiologist.

### Outcome Measures

#### Primary Outcome Measure

The primary outcome measure was tinnitus severity as measured by the TFI [43]. A meaningful change was defined to occur when scores were reduced by ≥13 points [43]. The TFI has been translated into >15 languages and has been validated for several populations, including the Chinese, Dutch, Swedish, and German [44]. It was selected over other tinnitus questionnaires as it was specifically developed to measure tinnitus severity and assess responsiveness to treatment and for comparison with previous trials [22-25].

#### Secondary Outcome Measures

Secondary outcome measures assessed anxiety [45], depression [46], insomnia [47], tinnitus cognitions [48], general health-related quality of life [49], and a short measure of hearing-related difficulties [50] as shown in Table 1. All questionnaires were used with the required permissions, and agreements were set up for those that were not freely available to use. For Spanish speakers, validated Spanish-translated versions were used. Where these were unavailable, validated translations were undertaken [39].

### Weekly Monitoring of Tinnitus During the Active Intervention Period

Throughout the program, tinnitus in participants was monitored weekly using the Tinnitus Handicap Inventory–Screening (THI-S) version. The THI-S consists of a 10-item questionnaire, and scores are comparable (*r*=0.90) with the full version of the Tinnitus Handicap Inventory (THI) [37]. Weekly scores were also used to detect possible adverse effects. If scores increased by more than 10 points between 2 consecutive weeks, this was noted as an adverse effect. Those indicating adverse effects were contacted to address the identified problems. Participants were also monitored using a newly developed Tinnitus Qualities Questionnaire (TQQ) [21]. The TQQ measures tinnitus qualities such as pitch, loudness, and the number of tones heard. The TQQ scores can range from 0 to 100, with higher scores indicating more problematic tinnitus.

### Intervention Variables

Intervention compliance was assessed by determining the retention rates and compliance in completing the outcome questionnaires. Intervention engagement was assessed by the number of log-ins, the number of modules read, and the number of messages sent during the intervention. Adverse effects were monitored by (1) direct questioning in the outcome questionnaire regarding the presence of adverse effects, (2) adverse effects written in messages or worksheets, and (3) an increase of 10 points or more during weekly monitoring using the THI-S questionnaire.

### Questionnaire Administration

Web-based questionnaires were used throughout the study for both groups. Although not all measures were validated for web-based use, the results were to be comparable, as equivalent psychometric properties have been reported [51]. All the measures were completed at baseline (T0), T1 (after the intervention for experimental group), T2 (after the intervention for the control group) and at the 2-month follow-up for the experimental group). To measure the control group 2 months after the intervention, participants from the control group completed further outcome measures at T3 (adjusted). For data analysis, the T3 results for the control group and those at T2 for the experimental group were compared to assess the invention effect at the same experimental time point for both groups (ie, 2 months after the intervention). To maximize retention, 3 electronic reminders were sent to participants who had not completed questionnaires on 3 consecutive days after the release of the questionnaire. A further reminder was sent via email and text messages. If questionnaires were not completed, participants were telephoned to encourage completion of the questionnaire. Participants were also telephoned after completing the intervention to discuss the progress they had made and share their questionnaire results.

### Statistical Analysis Plan

Statistical analyses were performed using the SPSS (version 26.0). All statistical tests were 2-tailed with an α set to .05. To account for missing data from participants not completing the postintervention or follow-up intervention analysis, an imputation analysis was undertaken. As the data were missing at random, missing data were handled through multiple imputation using the Markov Chain Monte Carlo approach owing to its ability to reduce bias even when the proportion of missing data was large [52,53]. For comparison, a complete case analysis was also performed by analyzing only the completed questionnaire data without imputing missing data. As there were substantial differences, statistical analysis using the imputed data is reported in the results section as a more accurate unbiased account of the findings.

The primary study outcome was a change in the TFI score among the groups at after the intervention (T1). A difference in scores between T1 and T2 for the experimental group was used to assess the stability of the intervention effects. Effect sizes, linear mixed effects models, and a reliable change index were used to assess the primary and secondary outcomes. Changes in baseline to postintervention scores were compared within and among the groups using the pre- and posttest effect sizes (Cohen *d*) for all primary and secondary outcomes using the observed data. Effect sizes of Cohen *d=*0.20 represent small effect sizes, those of Cohen *d*=0.50 represent medium effect sizes, and those Cohen *d*≥0.80 represent large effect sizes [54].

A linear mixed model, which provided unbiased results in the presence of missing data (using all available data), was applied to analyze the intervention effect over time for each outcome measure. An unstructured repeated effects and identify-random effects covariance structure provided the best model fit based on the Akaike Information Criterion. Time was treated as a repeated and fixed effect. A restricted maximum-likelihood estimation was applied. The Type III *F* test sums of squares from the linear mixed model were calculated. As a sensitivity analysis, baseline tinnitus severity was initially added as a covariate, but as it had no significant effect on the results, it was removed from the model.

Another model was run to test the differences during the course of the 8-week intervention for weekly tinnitus outcome measures. Posthoc time comparisons were carried out in the case of significant group differences to assess at which time points these differences occurred. In addition to statistical significance, clinical significance has also been reported. A 13-point difference is recommended by the original developers of the TFI [43] to indicate a meaningful change in scores. To handle study variability, the reliable change index [55] is recommended as a means of calculating clinical significance for the TFI as the primary outcome. This was calculated using the mean pretest–posttest score difference, the pretreatment SD (17.49), and a test–retest reliability coefficient of 0.78 as reported in the validation study.

### Sample Characteristics

Descriptive statistics including gender, age, ethnicity, race, tinnitus duration, hearing aid use, professional consultations, ease of computer use, veteran status, education, and employment status were used to describe the sample. The means and SDs were reported for each outcome measure at each time point. Descriptive statistics, including the number of log-ins and modules read, were also used to describe the sample and intervention engagement. A chi-square test of independence was used to identify group differences in engagement and compliance rates.

## Results

### Participant Characteristics

A total of 158 (50.2%) adults of the 315 participants screened met the eligibility criteria and were randomly assigned to the experimental (n=79) and control groups (n=79) as shown in Figure 1. In all, 8 (5.1%; 4 in each group) of the 158 participants were Spanish speakers who completed the Spanish version of the internet-based CBT program. Of the total sample (N=158), 80 participants (50.6%) were women and 78 participants (49.4%) were men with a mean age of 57 (SD 12) years for the full sample. The groups were well matched, and there were no clinically meaningful differences as seen in Table 2. Most participants (144/158, 91.1%) indicated that they were frequent users of computers and the internet. There were no functional failures related to the intervention during the trial. This trial commenced at the end of March 2020. This timing was unfortunate, as it coincided with the peak of the COVID-19 pandemic. Some participants reported becoming ill, struggling to adjust emotionally, or finding the required lifestyle changes difficult.

**Table 2:** Demographical characteristics of the participants.

Category and description		Experimental group (n=79)	Control group (n=79)	Overall (N=158)
**Gender, n (%)**				
	Male	40 (50.6)	38 (48.1)	78 (49.3)
	Female	39 (49.4)	41 (51.9)	80 (50.6)
Age (years), mean (SD; range)		56 (13; 19-76)	58 (11; 29-84)	57 (12; 19-84)
Tinnitus duration (years), mean (SD; range)		15 (16; 4 months to 70 years)	12 (12; 3 months to 58 years)	14 (14; 3 months to 70 years)
**Ethnicity, n (%)**				
	Hispanic or Latino	9 (11.4)	11 (13.9)	20 (12.7)
	Not Hispanic or Latino	70 (88.6)	68 (86.1)	138 (87.3)
**Race, n (%)**				
	American Indian or Alaska native	0 (0)	0 (0)	0 (0)
	Asian	1 (1.3)	0 (0)	1 (0.5)
	Native Hawaiian or Pacific Islanders	0 (0)	0 (0)	0 (0)
	Black or African American	2 (2.5)	2 (2.5)	4 (2.5)
	White	74 (93.7)	74 (93.7)	148 (93.7)
	More than 1 race	2 (2.5)	3 (3.5)	5 (3.1)
**Highest educational level, n (%)**				
	High school	11 (13.9)	10 (12.7)	21 (13.2)
	College or vocational training	22 (27.8)	31 (39.2)	53 (33.5)
	University degree	46 (58.2)	38 (48.1)	84 (53.2)
**Employment, n (%)**				
	Skilled or professional	55 (69.6)	41 (51.9)	96 (60.7)
	Retired	22 (27.8)	30 (38)	52 (32.9)
	Not working	2 (2.5)	8 (10.1)	10 (6.3)
**All professionals seen, n (%)**				
	Primary care physician	41 (51.9)	44 (55.7)	85 (53.7)
	Ear, nose, and throat physician	33 (41.8)	36 (45.6)	69 (43.7)
	Audiologist	36 (45.6)	39 (49.4)	75 (47.5)
**Veterans**				
	Veterans, n (%)	8 (10.1)	11 (13.9)	19 (12)
	Duration in the military service, mean (SD; range)	8 (3; 2-10)	8 (6; 2-23)	8 (5; 2-23)
**Ease of using a computer, n (%)**				
	Basic skills	7 (8.9)	7 (8.9)	14 (8.9)
	Frequent user	72 (91.1)	72 (91.1)	144 (91.1)

### Retention, Compliance, Engagement, and Adverse Effects

**Figure 2:**
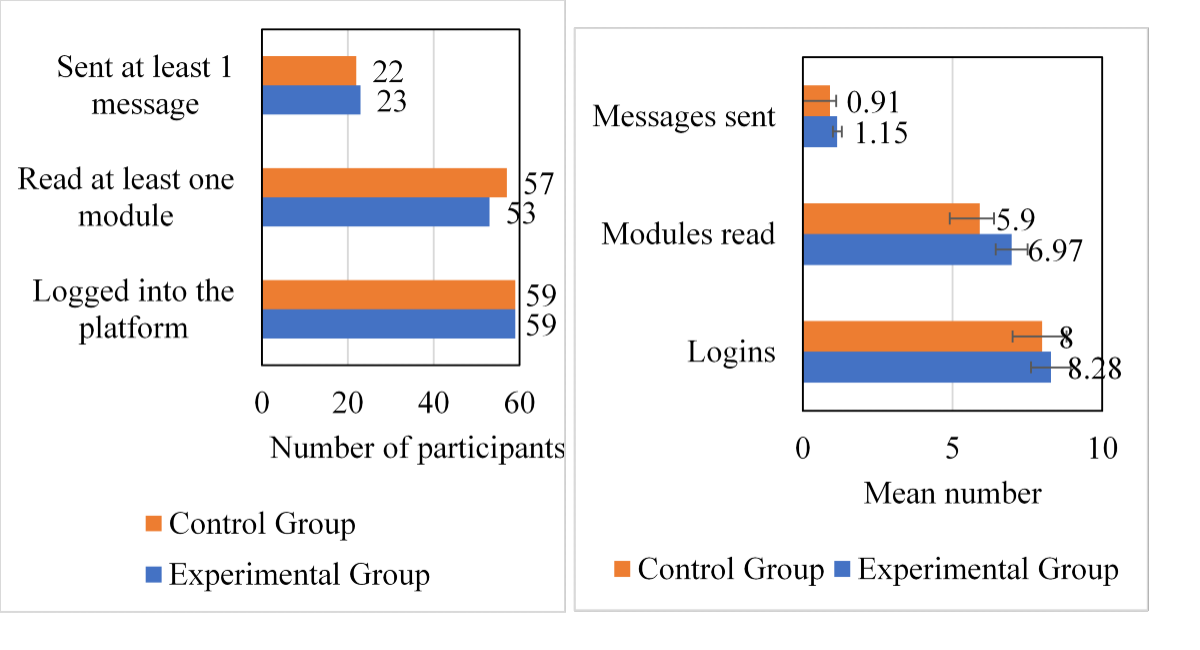
Intervention engagement.

Overall compliance for completing the outcome measures was low, with 57% (41 participants) and 51% (37 participants) completion rates at T1 and T2, respectively, for the experimental group (Figure 1). Although compliance was greater in the control group with 87% (66 participants) and 62% (47 participants) completion at T1 and T2, there was only 33% (25 participants) completion for the control group at T3, resulting in a significant difference between the group completion rates (*χ*^2^_3_=7.98; N=411; *P*=.046) at T3 with lower completion by the control group as shown in Figure 2.

Adverse effects have been reported to be low. During the intervention period, only 1 (0.6%) participant had an increase of more than 10 points in the THI-S questionnaire. On finding out more, this was related to a particularly stressful deadline for work under difficult circumstances during the COVID-19 pandemic. Only 1 (0.6%) participant reported an adverse effect on the outcome questionnaire, explaining that initially their tinnitus was more bothersome because of the focus on tinnitus at the start of the intervention. There were no serious adverse events, such as privacy breaches or major technical problems.

Intervention engagement was low but varied considerably among the participants. To identify if group allocation contributed, engagement among groups was compared, whereas each group was actively involved with the intervention. However, no significant group differences were identified (*χ*^2^_1_=0.13; N=273; *P*=.93) as shown in Figure 2. On average, 82% (59/72 participants) of the experimental group and 77% (59/76 participants) of the control group logged into the platform; 74% (53/72 participants) from the experimental group and 79% (57/72 participants) of the control group read at least one module, and 32% (23/72 participants) from the experimental group and 30% (23/76 participants) from the control group sent at least one message.

### Efficacy of Internet-Based CBT in Reducing Tinnitus Distress Compared With Weekly Monitoring

**Figure 3:**
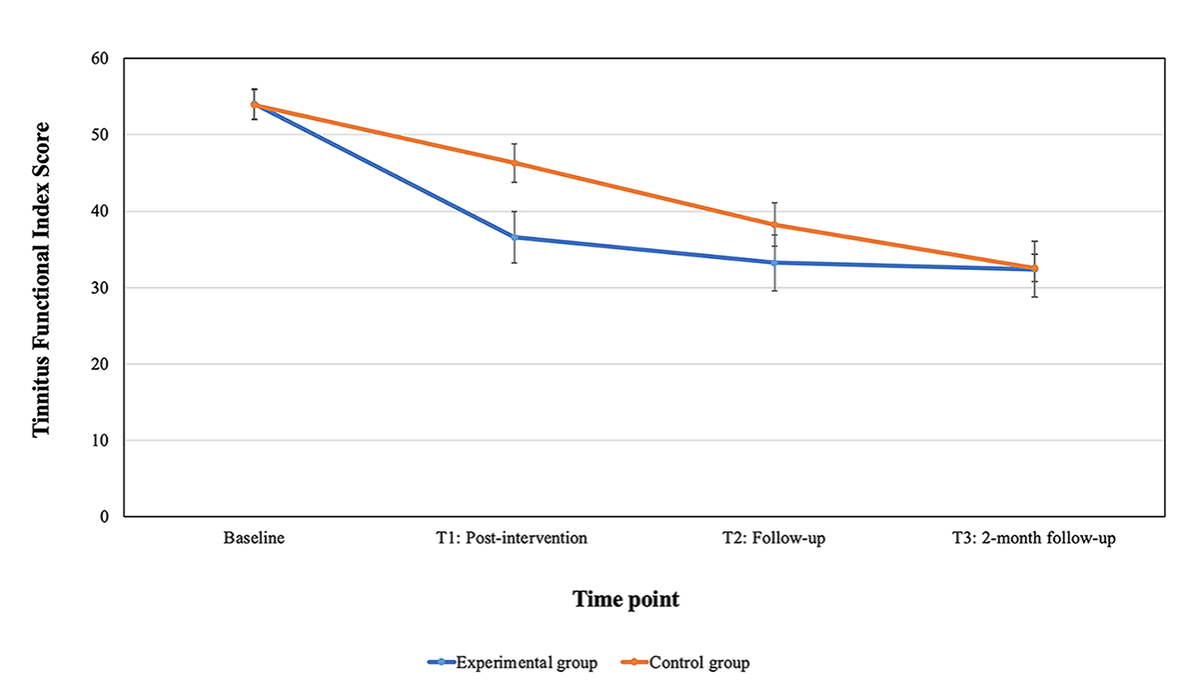
Change in tinnitus severity among groups over time. T1: only the experimental group had the intervention; T2: after the intervention for the control group and 2-month follow-up for the experimental group; T3: the comparison of the 2-month follow-up for both groups.

The tinnitus severity among the treatment arms was not constant over time (Figure 3 and Multimedia Appendix 2). The mean difference indicated a significant difference for the internet-based CBT group with an effect size of Cohen *d=*0.46 at T1. The test of fixed effects (Table 3) indicated that the intercept, slope, and group by time interaction had significant effects on the changes in tinnitus severity. There was no estimated difference in baseline tinnitus severity among the groups (*P*=.92). The posttreatment effect was significantly lower in the control group. After the experimental group underwent treatment (T1), they had an estimated 10-point decrease in tinnitus severity (CI 3-16; t_156.00_=−2.88; *P*=.004). After the control group also underwent treatment (T2), there was a nonsignificant estimated difference of 5 points (CI −2 to 11; t_156.00_=−1.48; *P*=.14). This may have been because of the initial large reduction (mean 7.62, SD 18.21 points) in scores during weekly monitoring despite not having the intervention.

**Table 3:** Random intercept mixed model results using results from the imputation data.

Outcome predictor	Intercept			Time			Group			Time × group	
	*F* test (*df*)	*P* value	*F* test (*df*)		*P* value	*F* test (*df*)		*P* value	*F* test (*df*)		*P* value
Tinnitus	1364.769 (1,156)	<.001	77.21 (3,156)		<.001	2.80 (1,156)		.10	3.64 (3,156)		.01
Anxiety	534.153 (1,156)	<.001	4.74 (3,156)		.003	0.05 (1,156)		.83	0.841 (3,156)		.47
Depression	489.593 (1,156)	<.001	12.250 (3,156)		<.001	0.05 (1,156)		.82	0.163 (3,156)		.92
Insomnia	637.397 (1,156)	<.001	42.064 (3,156)		<.001	1.81 (1,156)		.18	2.33 (3,156)		.08
EQ-5D-5L^a^	2034.549 (1,156)	.001	14.33 (3,156)		<.001	0.13 (1,156)		.72	0.19 (3,156)		.90
EQ-5D-5L visual analog scale	8100.537 (1,156)	<.001	2.02 (3,156)		.11	0.64 (1,156)		.43	1.63 (3,156)		.19
Tinnitus and Hearing Survey: tinnitus	294.231 (1,156)	<.001	38.850 (3,156)		<.001	0.750 (1,156)		.39	3.312 (3,156)		.02
Hearing disability	526.930 (1,156)	<.001	21.511 (3,156)		.001	2.24 (1,156)		.14	2.15 (3,156)		.10
Hyperacusis	247.016 (1,156)	<.001	2.51 (3,156)		.06	0.017 (1,156)		.90	0.410 (3,156)		.75
Tinnitus cognitions	1715.178 (1,156)	<.001	19.29 (3,156)		<.001	2.28 (1,156)		.13	4.15 (3,156)		.007

^a^EQ-5D-5L: European Quality of Life Five Dimension.

The model indicated an estimated baseline to 2-month follow-up mean difference of 22 points (CI 18-25) after undertaking the intervention with an estimated TFI score of 32 (baseline score was 54) at follow-up (CI 30-34).

**Figure 4:**
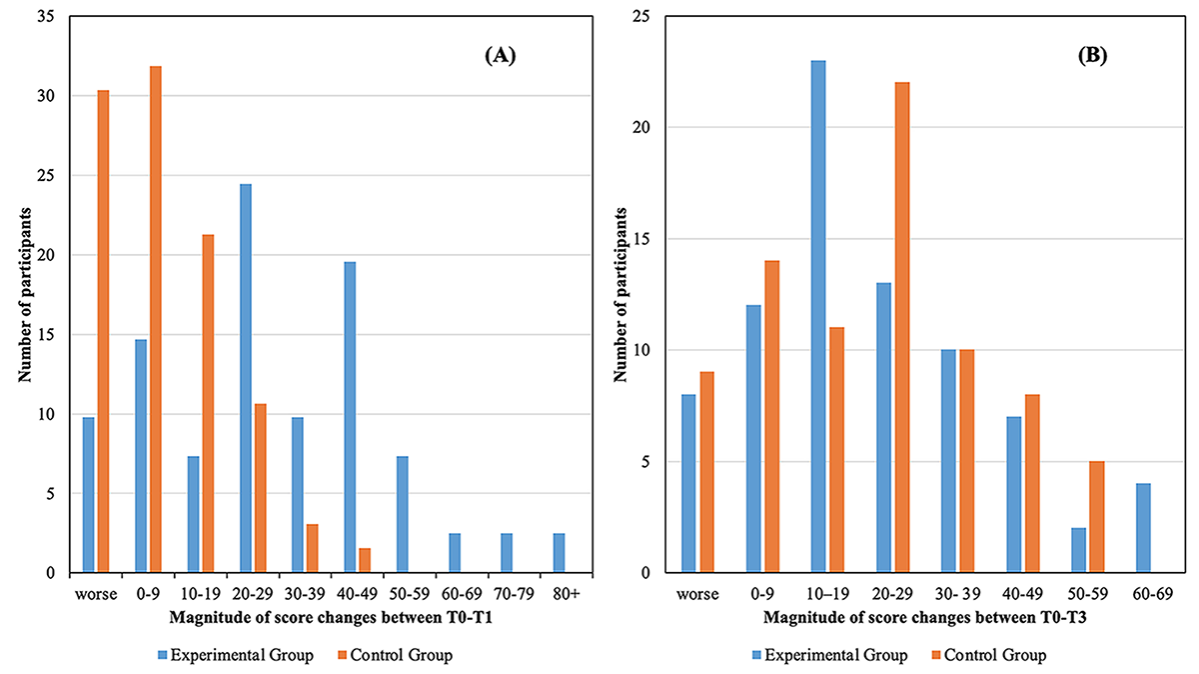
(A) Magnitude of Tinnitus Functional Index score changes between T0 and T1 after the experimental group underwent internet-based cognitive behavioral therapy. The control group was monitored weekly. (B) The magnitude of these changes between T0 and T3 at the 2-month follow-up after both groups completed the full intervention.

A comparison of the margin of score reduction between T0 and T1 is shown in Figure 4. This indicates that the experimental group had a greater score reduction (between 20 and 50 points) owing to the intervention with a maximum reduction of 88 points, compared with a maximum reduction of 44 points in the control group (with the majority between 0 and 9 points) who were only monitored weekly during this period.

The clinical significance was calculated using a reliable change index. The reliable change criterion was calculated to be 22.74 in TFI score. Using this value, clinical significance was achieved by 45 (57%) participants in the experimental group and 12 (15%) participants from the control group at T1 (after the intervention was performed in the experimental group). A clinically significant change was found in 40 (51%) participants of the experimental group at T2 (2 months after the intervention) and 30 (38%) participants of the control group (after the control group completed the intervention) and 40 (48%) participants of the control group at the 2-month follow-up.

### Efficacy of Internet-Based CBT in Reducing Tinnitus Comorbidities Compared With Weekly Monitoring

Results from the secondary assessment measures among the treatment arms were not constant over time, except for hyperacusis and the European Quality of Life Five Dimension visual analog scale scores for health-related quality of life, which did not have significant time effects (Table 3). After the intervention (T1), the experimental group had a significantly greater reduction in tinnitus cognition scores, indicating a medium effect (rounded off to Cohen *d*=0.50). The intercept, slope, and time by group interaction revealed significant effects on the changes in tinnitus cognition. Baseline tinnitus cognition was not significantly different among the groups (*P*=.58). After completing the intervention (T1), the experimental group had an estimated 8-point decrease in tinnitus cognition (CI 3-13; t_156_=−3.13; *P*=.002). After both groups undertook the intervention (T2), there was no significant estimated difference among the groups (*P*=.08). For tinnitus cognitions, the model indicated an estimated baseline to 2-month follow-up mean difference of 7 points (CI 3-11) after undertaking the intervention with an estimated score of 32 at follow-up (CI 31-32).

The experimental group had a significantly greater reduction in insomnia scores after the intervention (T1) and at follow-up (T2), although there was only a small effect (Cohen *d*=0.30) at T1 and medium effect (rounded off Cohen *d*=0.50) at T2. There was no significant group by time interaction indicated by the test of fixed effects owing to later improvements by the control group. Likewise, although the experimental group had a significantly greater reduction in hearing disability at T1, this was only a small effect (Cohen *d*=0.3) without a significant group by time interaction owing to later improvements by the control group.

Confirming the results of the primary outcome, the Tinnitus and Hearing Survey secondary measure indicated that the experimental group had a significantly greater reduction in tinnitus (Cohen *d*=0.60) with no significant difference at baseline or after the control group completed the intervention. The test of fixed effects results indicated that there was no significant time by group interaction for anxiety, depression, health-related quality of life, or hyperacusis outcome measures, and no significant effect was seen.

### Stability of Internet-Based CBT Intervention Effects 2 Months After the Intervention

At the 2-month follow-up, the experimental group indicated further reduction in tinnitus severity and all other outcomes. There were no significant differences in scores between T1 and T2 for the experimental group, indicating that intervention effects were maintained 2 months after the intervention.

At the 2-month follow-up, most of the secondary outcome measure scores were stable. However, there was an increase in anxiety scores for both groups, an increase in negative tinnitus cognitions for the experimental group, and a decrease in health-related quality of life for the control group, although these differences were not statistically significant.

### Comparison of Weekly Tinnitus Severity During the Active Intervention Period

**Figure 5:**
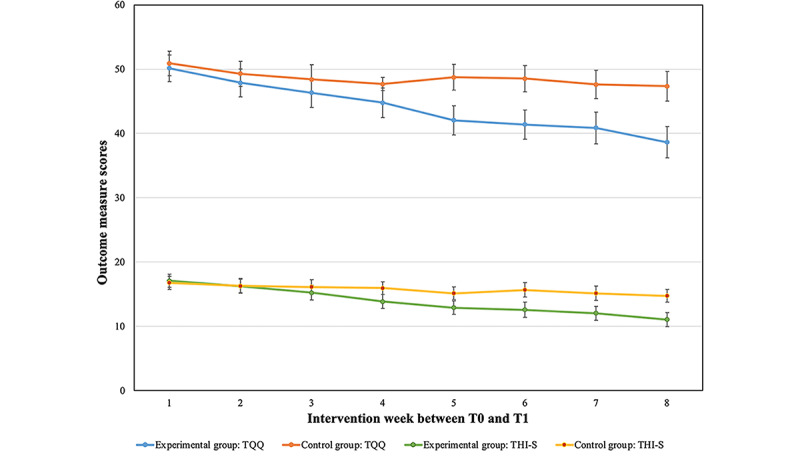
Weekly monitoring between the experimental and control group between T0 and T1. THI-S: Tinnitus Handicap Inventory–Screening; TQQ: Tinnitus Qualities Questionnaire.

Differences among the intervention arms were not constant across the eight time points between T0 and T1 for both the THI-S and TQQ outcome measure scores. The experimental group had a greater weekly reduction in tinnitus distress as evidenced by the significant group by time interaction for both the THI (*F*_7,136.000_=4.02; *P*=.04) and TQQ (*F*_7,136.000_=2.55; *P*=.02), as well as a significant intercept and slope. Pairwise comparisons indicated significant differences among groups from week 5 for the TQQ and week 6 for the THIs, as the experimental group’s (receiving internet-based CBT) tinnitus distress was rated significantly lower than that of the control group (not undergoing internet-based CBT). The maximum between-group mean difference in scores was at week 8, with the experimental group having a THI-S score of 3.5 (SE 1.1) points lower and TQQ score of 8.72 (SE 2.30) lower than that of the control group, as seen in Figure 5.

## Discussion

### Overview

This study is the first to evaluate the efficacy of an audiologist-delivered internet-based CBT in reducing tinnitus distress in a US population. It was also the first study to offer internet-based CBT in Spanish to accommodate the Hispanic population in the United States. The study objectives were to evaluate the efficacy of the audiologist-delivered internet-based CBT in reducing tinnitus distress and the comorbidities associated with tinnitus compared with that of weekly monitoring of tinnitus. Furthermore, the stability of the intervention effects was assessed 2 months after the intervention.

### Principal Findings

Participating in the internet-based CBT intervention led to significantly greater improvements in tinnitus distress and a medium effect size compared with that of weekly monitoring. This adds to the evidence base regarding the feasibility of audiologist-guided internet-based CBT as indicated in clinical trials in the United Kingdom using audiologist guidance [22-25]. These results are also in line with earlier internet-based CBT trials for tinnitus, indicating a pooled medium effect size (Cohen *d*=0.59) (for a review see the study by Beukes et al [28]) with those from Europe using psychologist guidance.

Most participants had a reduction of between 20 and 50 points in their TFI scores, although a range of outcomes was observed. Improvements were found in the control group after weekly monitoring, which has also been previously mentioned [56]. This may also be the effect of the control group knowing that they will be receiving an intervention, and this expectation may have helped them manage better before receiving the actual intervention. This initial improvement did seem to affect the postintervention results, which were lower in the control group than in the experimental group. When comparing the participants in each group on a weekly basis, it was observed that the experimental group had a greater reduction in tinnitus distress over the 8-week period. Significant differences were present from week 5 for TQQ and week 6 for THI. This was slightly later in the intervention than was found in the United Kingdom trial when comparing differences for the THI by week 4 [23]. After the control group undertook the intervention, they made similar significant improvements as those demonstrated by the experimental group, and no significant differences were found among the groups. These results were maintained at the 2-month follow-up for both groups, although the magnitude of reduction was more variable, with the most participants from the experimental group indicating a 10- to 19-point reduction and those from the control group, a 20- to 29-point reduction in TFI scores. Further studies are required to assess whether they are maintained long-term (eg, 1 year) as has been found by previous internet-based CBT for tinnitus trials [17,25,57,58].

More participants from the experimental group had a clinically significant difference (57%; 45 participants) after their treatment compared with 38% (30 participants) of the control group following their treatment. At the 2-month follow-up, 51% (40 participants) and 48% (40 participants) from each group achieved a clinically significant change in tinnitus distress. This was lower than for the pilot study [32] owing to the reliable change criterion required being higher for this study because of a larger baseline SD. As the reliable change criterion was similar to internet-based CBT efficacy trial in the United Kingdom [23], comparable proportions of participants reached clinical significance in this study.

### Secondary Results

Experiencing tinnitus is accompanied by various comorbidities that may exacerbate distress and negative emotional responses to perception. An important aspect of tinnitus intervention is the ability to address these challenging comorbidities. Undergoing internet-based CBT resulted in a significantly greater reduction in negative tinnitus cognitions (Cohen *d*=0.46) and insomnia (small effect). This finding is in line with the pooled results from previous internet-based CBT studies, also indicating a small effect (Cohen *d*=0.42). This was the first internet-based CBT for tinnitus study that included the tinnitus cognition questionnaire. The finding that this CBT intervention is able to reduce negative thought patterns associated with tinnitus is a positive finding and continued use of the tinnitus cognition questionnaire is important as recommended by Handscomb et al [59]. From the pooled results of internet-based CBT studies, there was no effect on quality of life [28], similar to this study, but there was a greater reduction in anxiety and depression, which was not found in this study. The reason may be related to the exclusion of individuals with severe mental health conditions, possibly reducing the opportunity to observe an intervention effect owing to the low baseline scores. Broader inclusion criteria are necessary to ensure internet-based CBT is provided to all affected individuals, including those with mental health conditions, as they seem to benefit from this as shown in previous studies [17,19,21-25,57,58]. The pilot study also indicated an effect for hearing disability and hyperacusis [32], which was not observed in this study. The content addressed in the modules providing hearing tactic strategies and advice on reducing sound sensitivity were in the optional modules, which were not read by many participants, and this may have contributed to these results. Interestingly, changes were noted in the TQQ, suggesting that the internet-based CBT may result in a change in tinnitus perception (eg, tinnitus pitch, loudness, and number of sounds heard) in addition to a reduction in tinnitus distress. However, these observations should be replicated in future studies, in addition to possible biomarkers.

### Comparison With Previous Work

The study participants’ characteristics were similar to those found in previous internet-based CBT trials; however, the mean age was slightly higher at 57 years than at a mean age of 51 years [28]. Despite extensive recruitment strategies and campaigns, following suggestions to support Hispanic and Latino research participants [60], only 8 participants selected to participate in the intervention in Spanish. Further efforts will be required to build trust within Hispanic communities before recruiting for subsequent trials [61].

Although the study administration mimicked that that would be provided in a routine application, the overall completion rate of the posttreatment questionnaires was low across time points and groups, compared with that of previous internet-based CBT for tinnitus studies [28]. Although only 10 participants (6%) withdrew, many enrolled participants never logged into the intervention website. Moreover, not all the modules were read, and very few messages were sent, indicating low intervention engagement. Numerous factors contribute to this finding. One may be the timing of this study during the COVID-19 pandemic. Participants explained that they were on their computers all day attending meetings on Zoom as they had to stay at home. This may be an additional computer work, making this intervention difficult, because of them wanting to take a break from their computers. Some participants mentioned having contracted the COVID-19 virus, and even after recovering, they remained fatigued, making intervention engagement difficult. Others found the lifestyle changes of working from home and juggling childcare difficult, and some were affected emotionally. However, the COVID-19 pandemic is unlikely to be the only reason for poor engagement. Differences in compliance among groups were also observed. After receiving the intervention, compliance was lower in the experimental group and lower in the control group at the 2-month follow-up. This may have reflected the trial design that the control group had an additional assessment time point and having already completed assessments after the control and intervention, decided not to complete another assessment at the 2-month follow-up. During the pilot study [32], lower engagement than noted in earlier studies was observed. Cultural differences may not be accounted for. In contrast to the United Kingdom and many parts of mainland Europe, many people pay for health care via third-party reimbursements in the United States. The internet-based CBT intervention was offered free of charge. It may be that people undervalued this treatment as a free treatment that may be perceived as less effective than one requiring payment. In addition, this study recruited participants only from Texas, United States, thus representing a very small population of the United States and may not represent the wider US population. Subsequent trials should be performed with a wider US population. Process evaluation may be helpful in identifying the factors contributing to the retention and engagement rates and identifying what may improve theses [26]. Continued public involvement in planning and implementing subsequent research trials will be vital to gain insights into the factors important to participants [62,63].

### Limitations

This study represents participants living in Texas, United States, who do not present with severe mental health conditions, often associated with tinnitus. This may not present the typical tinnitus population, and thus, the findings cannot be generalized to other populations. Despite recruitment efforts, only 8 Spanish participants were recruited; similarly, the participant groups also contained low numbers of ethnic and racial minorities when compared with the general population from this region. The study was furthermore conducted during the peak of the COVID-19 pandemic, a time when day-to-day living was disrupted for most people. The results may also have been different if a large proportion of participants were engaged and completed the outcome assessments.

### Conclusions

Compounding the potentially debilitating nature of severe tinnitus, accessible, evidence-based interventions are still lacking. There is an urgent need to improve the availability of such interventions. Furthermore, the COVID-19 pandemic has highlighted the need for evidence-based eHealth approaches to overcome the limited in-person contact and support available for individuals with tinnitus [63]. These results further support the role of audiologists in guiding such forms of tinnitus management. The results have been encouraging, and further work is indicated in view of making such an intervention applicable to a wider population. Future work should consider enrolling heterogeneous tinnitus populations to examine who are more suitable (or not) for the internet-based CBT program. In addition, a stepped approach (eg, a brief intervention offered to all participants and a comprehensive intervention for more suitable patients following the brief intervention) should be examined to improve compliance and engagement.

## Appendix

Multimedia Appendix 1 CONSORT-eHEALTH (Consolidated Standards of Reporting Trials of Electronic and Mobile Health Applications and Online Telehealth) checklist (V 1.6.1).

Multimedia Appendix 2 Outcome measures at each time point.

## Abbreviations

CBT: cognitive behavioral therapy
CONSORT-EHEALTH: Consolidated Standards of Reporting Trials of Electronic and Mobile Health Applications and Online Telehealth
RCT: randomized controlled trial
TFI: Tinnitus Functional Index
THI: Tinnitus Handicap Inventory
THI-S: Tinnitus Handicap Inventory–Screening
TQQ: Tinnitus Qualities Questionnaire
