# Supplementary material for: Internet-Based Audiologist-Guided Cognitive Behavioral Therapy for Tinnitus: Randomized Controlled Trial
Source: J Med Internet Res. 2022 Feb 14;24(2):e27584. doi: 10.2196/27584 (PMC8887633; doi:10.2196/27584)
Supplement: Multimedia Appendix 2 [file jmir_v24i2e27584_app2.docx]

**Multimedia Appendix 2.** Outcome measures at each time point.

| Outcome measure and group allocation | | | | | T0 (pretreatment measurements at baseline) | T1 (experimental group: postintervention measurements; control group: postweekly monitoring) | T2 (experimental group: follow-up for; control group: posttreatment measurements) | T3 (experimental group: repeated follow-up, not measured; control group: follow-up) | Between-group Cohen *d* (95% CIs) |
| --- | --- | --- | --- | --- | --- | --- | --- | --- | --- |
| **Primary outcome; mean (SD)** | | | | | | | | | |
|  | **Tinnitus Functional Index** | | | | | | | | *At T1, 0.46 (0.14 to 0.77)*; at T2, 0.23 (−0.09 to 0.54); at T3; 0.02 (−0.30 to 0.33) |
|  |  | | | Experimental group | 54.04 (17.85) | Completers: 29.44 (19.60); Imputation: 36.57 (22.00) | Completers: 25.10 (19.46); Imputation: 33.24 (22.00) | Completers: 25.10 (19.46); Imputation: 32.39 (14.70) |  |
|  |  | | | Control group | 53.93 (17.32) | Completers: 47.35 (21.25); Imputation: 46.31 (20.63) | Completers: 38.50 (21.36); Imputation: 38.25 (21.24) | Completers: 32.82 (26.80); Imputation: 32.57 (15.37) |  |
| **Secondary outcomes; mean (SD)** | | | | | | | | | |
|  | | **Anxiety (Generalized Anxiety Disorder-7)** | | | | | | | At T1, 0.00 (−0.31 to 0.31); at T2, 0.00 (−0.31 to 0.31); at T3, 0.12 (−0.20 to 0.43) |
|  | |  | | Experimental group | 5.97 (4.22) | Completers: 4.80 (4.84); Imputation: 5.67 (4.71) | Completers: 3.77 (4.65); Imputation: 4.63 (4.34) | Completers: 3.77 (4.65); Imputation: 5.07 (2.43) |  |
|  | |  | | Control group | 5.23 (4.29) | Completers: 5.35 (4.59); Imputation: 5.68 (4.73) | Completers: 3.77 (3.52); Imputation: 4.62 (4.03) | Completers: 5.17 (5.58); Imputation: 5.43 (3.66) |  |
|  | | **Depression (PHA-9)** | | | | | | | At T1, 0.07 (−0.24 to 0.38); at T2, 0.07 (−0.24 to 0.38); at T3, −0.004 (−0.35 to 0.28) |
|  | |  | | Experimental group | 5.54 (4.17) | Completers: 4.41 (4.74); Imputation: 5.72 (4.93) | Completers: 3.17 (4.01); Imputation: 5.11 (4.86) | Completers: 3.17 (4.01); Imputation: 3.87 (2.01) |  |
|  | |  | | Control group | 5.42 (4.10) | Completers: 5.91 (4.35); Imputation: 6.04 (4.23) | Completers: 4.20 (3.80); Imputation: 5.44 (4.03) | Completers: 3.70 (4.73); Imputation: 3.78 (2.96) |  |
|  | | **Insomnia (Insomnia Severity Index)** | | | | | | | *At T1, 0.34 (0.02 to 0.65); at T2 0.45 (0.13 to 0.76)*; at T3, 0.00 (−0.31 to 0.31) |
|  | |  | | Experimental group | 10.18 (5.99) | Completers: 5.98 (4.80); Imputation: 7.97 (5.43) | Completers: 4.97 (5.00); Imputation: 6.70 (5.55) | Completers: 4.97 (5.00); Imputation: 5.67 (2.18) |  |
|  | |  | | Control group | 9.92 (5.71) | Completers: 9.97 (6.39); Imputation: 9.97 (6.30) | Completers: 7.70 (5.56); Imputation: 8.24 (5.77) | Completers: 5.77 (6.24); Imputation: 5.81 (3.86) |  |
|  | | **Health-related quality of life (EQ-5D-5L)** | | | | | | | At T1, 0.07 (−0.24 to 0.39); at T2, 0.10 (−0.21 to 0.42); at T3, 0.05 (−0.27 to 0.36) |
|  | |  | | Experimental group | 7.44 (2.43) | Completers: 7.17 (2.05); Imputation: 7.38 (2.24) | Completers: 6.56 (1.52); Imputation: 6.72 (1.84) | Completers: 6.56 (1.52); Imputation: 6.88 (2.86) |  |
|  | |  | | Control group | 7.42 (2.48) | Completers: 7.50 (2.37); Imputation: 7.55 (2.34) | Completers: 6.78 (1.62); Imputation: 6.91 (1.80) | Completers: 7.00 (2.25); Imputation: 7.00 (2.35) |  |
|  | | **Health-related quality of life (EQ-5D-5L) visual analog scale scores** | | | | | | | At T1, 0.14 (−0.17 to 0.45); at T2, 0.23 (−0.09 to 0.54); at T3, −0.15 (−0.46 to 0.16) |
|  | |  | Experimental group | | 78.23 (13.14) | Completers: 79.68 (15.66); Imputation: 78.44 (14.83) | Completers: 80.79 (16.16); Imputation: 79.39 (14.40) | Completers: 80.79 (16.16); Imputation: 74.00 (15.56) |  |
|  | |  | Control group | | 75.51 (13.96) | Completers: 76.02 (13.70); Imputation: 76.43 (13.63) | Completers: 78.52 (13.29); Imputation: 76.21 (13.78) | Completers: 78.05 (21.44); Imputation: 76.48 (17.51) |  |
|  | | **Tinnitus score from the Tinnitus and Hearing Survey** | | | | | | | *At T1, 0.64 (0.32 to 0.96)*; at T2, 0.24 (−0.07 to 0.55); at T3, −0.06 (−0.37 to 0.25) |
|  | |  | | Experimental group | 5.80 (3.90) | Completers: 2.68 (2.47); Imputation: 3.34 (2.82) | Completers: 2.35 (3.40); Imputation: 2.73 (3.02) | Completers: 2.35 (3.40); Imputation: 3.75 (4.79) |  |
|  | |  | | Control group | 5.51 (3.52) | Completers: 5.31 (3.82); Imputation: 4.82 (4.02) | Completers: 3.46 (3.10); Imputation: 3.44 (2.86) | Completers: 2.86 (3.68); Imputation: 3.51 (3.50) |  |
|  | | **Hearing disability (Tinnitus and Hearing Survey)** | | | | | | | *At T1, 0.34 (0.02 to 0.65)*; at T2, 0.28 (−0.03 to 0.59); at T3, −0.11 (−0.42 to 0.20) |
|  | |  | Experimental group | | 6.89 (4.50) | Completers: 3.83 (3.65); Imputation: 5.00 (4.48) | Completers: 3.21 (3.20); Imputation: 4.80 (3.78) | Completers: 3.21 (3.20); Imputation: 4.29 (1.86) |  |
|  | |  | Control group | | 7.30 (4.79) | Completers: 7.29 (4.94); Imputation: 6.62 (5.15) | Completers: 6.46 (4.05); Imputation: 5.94 (4.28) | Completers: 4.14 (3.37); Imputation: 4.05 (2.38) |  |
|  | | **Hyperacusis (Tinnitus and Hearing Survey)** | | | | | | | At T1, 0.09 (−0.22 to 0.40); at T2, 0.09 (−0.22 to 0.40); at T3, −0.006 (−0.38 to 0.25) |
|  | |  | Experimental group | | 0.87 (1.3) | Completers: 0.54 (0.84); Imputation: 0.97 (1.02) | Completers: 0.47 (0.79); Imputation: 0.81 (0.77) | Completers: 0.47 (0.79); Imputation: 0.82 (0.71) |  |
|  | |  | Control group | | 0.80 (1.10) | Completers: 1.00 (1.30); Imputation: 1.07 (1.25) | Completers: 0.76 (1.14); Imputation: 0.89 (0.98) | Completers: 0.64 (.90); Imputation: 0.77 (0.83) |  |
|  | | **Tinnitus cognitions (Tinnitus Cognitions Questionnaire)** | | | | | | | *At T1, 0.46 (0.14 to 0.77*^a^*)*; at T2, 0.25 (−0.06 to 0.56); at T3, −0.03 (−0.34 to 0.28) |
|  | |  | Experimental group | | 39.63 (17.38) | Completers: 26.03 (15.58); Imputation: 34.17 (20.08) | Completers: 26.03 (16.80); Imputation: 29.60 (15.29) | Completers: 26.03 (16.80); Imputation: 32.29 (4.49) |  |
|  | |  | Control group | | 37.95 (14.54) | Completers: 40.57 (15.72); Imputation: 42.43 (15.75) | Completers: 32.26 (15.54); Imputation: 33.53 (16.12) | Completers: 28.55 (17.00); Imputation: 32.06 (9.54) |  |

a
